# A reproducible brain tumour model established from human glioblastoma biopsies

**DOI:** 10.1186/1471-2407-9-465

**Published:** 2009-12-29

**Authors:** Jian Wang, Hrvoje Miletic, Per Ø Sakariassen, Peter C Huszthy, Hege Jacobsen, Narve Brekkå, Xingang Li, Peng Zhao, Sverre Mørk, Martha Chekenya, Rolf Bjerkvig, Per Ø Enger

**Affiliations:** 1 Department of Biomedicine, University of Bergen, N-5009 Bergen, Norway; 2 Department of Pathology, University of Bergen, N-5021 Bergen, Norway; 3 Qilu Hospital, Shandong University, 250013 Jinan, China; 4 NorLux Neuro-Oncology, Centre Recherche de Public Santé, L-1150, Luxembourg; 5 Department of Neurosurgery, Haukeland University Hospital, N-5021 Bergen, Norway

## Abstract

**Background:**

Establishing clinically relevant animal models of glioblastoma multiforme (GBM) remains a challenge, and many commonly used cell line-based models do not recapitulate the invasive growth patterns of patient GBMs. Previously, we have reported the formation of highly invasive tumour xenografts in nude rats from human GBMs. However, implementing tumour models based on primary tissue requires that these models can be sufficiently standardised with consistently high take rates.

**Methods:**

In this work, we collected data on growth kinetics from a material of 29 biopsies xenografted in nude rats, and characterised this model with an emphasis on neuropathological and radiological features.

**Results:**

The tumour take rate for xenografted GBM biopsies were 96% and remained close to 100% at subsequent passages *in vivo*, whereas only one of four lower grade tumours engrafted. Average time from transplantation to the onset of symptoms was 125 days ± 11.5 SEM. Histologically, the primary xenografts recapitulated the invasive features of the parent tumours while endothelial cell proliferations and necrosis were mostly absent. After 4-5 *in vivo *passages, the tumours became more vascular with necrotic areas, but also appeared more circumscribed. MRI typically revealed changes related to tumour growth, several months prior to the onset of symptoms.

**Conclusions:**

*In vivo *passaging of patient GBM biopsies produced tumours representative of the patient tumours, with high take rates and a reproducible disease course. The model provides combinations of angiogenic and invasive phenotypes and represents a good alternative to *in vitro *propagated cell lines for dissecting mechanisms of brain tumour progression.

## Background

Valid model systems are a basic premise for clinical translation of experimental data. In tumour biology, model systems typically involve cancer cells that have been passaged numerous times *in vitro *to provide cancer cell lines [1]. In animals, the cells are often implanted subcutaneously, or in other ectopic locations [2] to produce experimental tumours. The fact that these models are easily established and standardized, favour their use in high throughput analyses. Although cell line models provide highly reproducible results, these findings may have a limited clinical relevance [3,4]. Notably, only a fraction of the drug candidates that show promising results in animal studies is ever implemented in treatment protocols for cancer patients [5-8]. Thus, more powerful tools than the models that are currently used are needed.

Orthotopic models are characterised by tumour growth in the animal, in a site corresponding to the human organ in which the tumour arises [9-11]. Therefore, such models provide an optimal system for studying tumour-host interactions, within an organ-specific niche [12,13]. Moreover, engrafting primary tissue instead of cancer cell lines may prevent a genetic drift and phenotypic changes induced by prolonged passaging *in vitro *[14]. Indeed, orthotopic tumour models, using primary tissue, have been established for several cancer subtypes [15-18], and studies have demonstrated that they can recapitulate tumour-host interactions and display traits of the parent tumour [19].

Previously, we established an orthotopic biopsy-based model to study angiogenesis and malignant progression [20]. These experiments demonstrated that engraftment of GBM biopsies produced highly infiltrative gliomas that could grow in the absence of angiogenesis, and that passaging was accompanied by the onset of angiogenesis and shorter survival. Thus, the resulting tumours mimicked essential phenotypic traits of GBMs in patients.

However, experimental tumour models must be reproducible as well as representative. Since orthotopic models are technically challenging, and since primary tumour tissue display more cellular heterogeneity than cell lines, their use has been limited. Therefore, data demonstrating sufficiently high take rates and a reproducible disease course is needed before we can implement these models to their full potential. In order to address these issues, we studied tumour growth in a consecutive series of 29 glioma biopsies used in xenograft implantations. The animals were scanned using MRI, survival was registered, and brains were examined. This series also included the xenograft tumours we previously used to investigate the invasive and vascular phenotypes in brain tumours [20]. However, the present work also provides experimental data about the growth parameters, neuropathological and radiological characteristics of these tumours.

## Methods

### Tissue culture

Tumour biopsy tissue was obtained from patients at the neurosurgical department at Haukeland University Hospital, Bergen. The study was approved by the regional Ethical Board at Haukeland University Hospital. Spheroids were prepared as described previously [14].

### Animal experiments

Studies were conducted with 197 male and female homozygous nude rats (Han:rnu/rnu Rowett) bred and maintained in an isolation facility in a pathogen free environment on a standard 12/12 h day and night cycle. Animals were fed a standard sterilised pellet diet and provided sterile tap water *ad libitum*. The athymic nude rat is T-cell deficient, but has normal complement and B-cell function [11]. 10 tumour spheroids (250-350 μm in diameter) were selected under a light microscope. The animals were anaesthetized with Hypnorm-Dormicum (0.4 ml/kg) s.c., the head secured in a stereotactic frame (Benchmark; Neurolab, St Louis, MO) and a short longitudinal incision was made in the scalp exposing the calvarium. A burr-hole was made 1 mm posterior to the bregma and 3 mm to the right of the sagittal suture using a micro-drill with a bit diameter of 2,9 mm. A Hamilton syringe with inner diameter of 810 μm was introduced to a depth of 2, 5 mm below the brain surface, and the spheroids were slowly injected and the syringe left in place for 3 min before withdrawal. The skin was closed with an Ethilon 3-0 suture. The tumours were allowed to grow for 4-5 months, then harvested and passaged onto new animals after initiation of spheroids *in vitro*. Animals were sacrificed at the onset of symptoms using CO_2 _and the brains were removed. All procedures and experiments involving animals in this study were approved by The National Animal Research Authority and conducted according to the European Convention for the Protection of Vertebrates Used for Scientific purposes.

### MRI Scanning

Patient MRI images were obtained by using a MRI Magnetom Vision Plus 1.5 T scanner (Siemens, Erlangen, Germany). Animals engrafted with tissue from the initial 9 biopsies were imaged, using the same MRI combined with a small circular finger coil [21]. Subsequently, animal images were obtained, using a Bruker Pharmascan 7T MR scanner (Bruker Biospin MRI GmbH, Ettlingen, Germany), an axial T1 weighted MSME sequence (TR 1000 ms, TE 8.7 ms, slice thickness 1 mm, FOV 3.5 cm, matrix size 256 × 256, 20 slices) was acquired before and after administration of subcutaneous injection of contrast agent (1.0 mL of 0.5 mmol/mL Omniscan; Nycomed Amersham, Oslo, Norway). An axial T2 weighted RARE sequence was also acquired (TR 4200 ms, TE 36 ms, slice thickness 1 mm, FOV 3.5 cm, and matrix size 256 × 256, 20 slices). During scanning, the animals were anesthetized with 1.5% isofluorane mixed with 50% air and 50% O2.

### Histological examination and immunohistochemistry

Brains were immersion fixed in 4% formaldehyde in Dulbecco's Phosphate-Buffered Saline for 24 h. The brains were then embedded in paraffin, and 5 μm sections were prepared. Every 20th section was collected for further histological analysis. These sections were stained with haematoxylin and eosin, and examined by a neuropathologist under a light microscope. Representative formalin-fixed, paraffin-embedded sections from each specimen were immunohistochemically stained with Ki67 (MIB-1, 1:500)) or human specific nestin (1:500) antibodies (Both Chemicon, Temecula, CA, US) by use of the ABC method and the DCS Detection-Kit with 3, 3"-Diaminobenzidine (DAB) and H_2_O_2 _(DCS, Hamburg, Germany). The fraction of labelled tumour cells, defined as the Ki67 labelling index (Ki67 LI), was assessed in 5 microscopic high power fields, appearing as "proliferative hot-spots", which contained a high fraction of labelled cells.

### Flow Cytometry

The cell cycle distribution of the tumours was determined by flow cytometric DNA analysis. The tumour tissue was minced, fixed in ice-cold 70% ethanol and washed in 0.9% NaCL and then incubated at 37°C for 15 min with 0.5% pepsin (Sigma, St Lois, MO) in 0.9% NaCl solution (pH1.5). The nuclear suspension was exposed to 100 μl of 1 mg/ml RNAse for 1 min, and then stained with propidium iodide (50 mg/ml, Sigma). All samples were filtered through a 60 mm nylon filter before analysis with a FACSort flow cytometer (Becton Dickinson, Palo Alto, CA). Gating was performed from a two-parameter intensity diagram (FL2 width/FL2 area) in order to exclude doublets and triplets within the fluid flow. Determination of the relative proportions of the cell cycle distribution was obtained using the FACS software platform.

### Tunnel staining

Tissue sections were de-paraffinised and the epitopes were unmasked with protease K (20 μg/ml, Sigma) for 10 min at room temperature. Detection of apoptotic cells was performed with the terminal deoxynucleotidyltransferase mediated nick end labelling (TUNNEL) assay according to the manufacturer's instructions (Roche Applied Bioscience, Manheim Germany). TUNNEL -positive cells were visualised with DAB as chromogen, and the fraction of Tunnel-positive cells was assessed from 5 high power fields.

### Statistics

A p-level of ≤ 0.05 was considered significant. We used the two-tailed Student's t-test for comparison and Spearmans' test to correlate culture time and patient age with survival time and take rates. We used log-rank test to analyse survival data. A p-level of ≤ 0.05 was considered significant.

## Results

### Patient characteristics and engraftment rates

We xenografted biopsies from 29 glioma patients, of which 25 were diagnosed with GBM, 3 patients had an astrocytoma grade II, and one had an oligoastrocytoma grade III (Table 1). None of the GBM patients had been diagnosed with a lower grade lesion prior to harvesting the GBM-biopsies, although 3 were recurrent GBMs (Case 4, 11 and 15). The age at the time of operation ranged from 23 to 81, 18 patients were males and 11 were females. The biopsy tissue was cut into 0.3 mm sized fragments that were cultured *in vitro *in agar-overlay cultures as previously described [14] and implanted as soon as spheroids had formed. The culture time ranged from 3 to 67 days, while the average culture time was 18 days ± 3.3 SEM days. 25 of 29 (86%) of the biopsies formed tumour spheroids during the first 4 weeks. Spheroids from the various biopsies were grafted onto 197 nude rats, and the number of animals receiving tissue from the same biopsy varied between 2 and 14. In total, 24 of the 25 GBM biopsies (96%) demonstrated a tumour take although at varying rates, as 137 of the 171 (80.1 ± 5.2 SEM%) animals xenografted with GBM biopsies developed tumours. Only one of the lower grade tumours engrafted in the nude rats (Gemistocytic astrocytoma, WHO grade II). The average time from implantation to onset of symptoms ranged from 58.5 to 326.5 days, with an average of 125 ± 11.5 SEM days. The longest time interval was recorded for the gemistocytic astrocytoma, 326.5 ± 20 SEM days. Amongst animals xenografted with GBM biopsies harvested at the primary operation, survival was 118 ± 9.3 SEM days. Although survival was shorter amongst animals grafted with recurrent GBMs (106 ± 9.9 SEM days), this difference was not significant (p = 0.61). Culture time correlated inversely with tumour take, but not with survival (p = 0.007 and p = 0.61, respectively). In addition, there was no correlation between patient age vs. tumour take or survival (p = 0.69 and p = 0.78, respectively).

**Table 1:** Overview of patient characteristics for the biopsies that were engrafted, engraftment rates and survival.

Case	Diagnosis	Gender	Age	Culture time, days	Tumour take, %	Survival, mean days ± SEM	Passage, yes/no
1	GBM	f	46	4	6/6 (100)	104.5 ± 1.4	yes
2	GBM	m	70	15	12/13 (92)	117.5 ± 8.6	yes
3	GBM	m	31	19	4/5 (80)	169.5 ± 22.1	no
4	GBM	f	31	4	7/7 (100)	97 ± 1.7	yes
5	GBM	m	70	23	8/8 (100)	119.5 ± 3.5	yes
6	GBM	m	45	24	2/10 (20)	93.5 ± 10.6	no
7	GBM	m	47	3	7/7 (100)	126.5 ± 2.9	yes
8	GBM	m	47	3	12/14 (86)	137.5 ± 5	no
9	GBM	m	62	6	3/5 (60)	252 ± 1.6	no
10	GBM	m	60	3	6/6 (100)	83 ± 6.6	no
11	GBM	m	38	7	3/3 (100)	94.5 ± 7.7	no
12	GBM	m	64	4	6/6 (100)	58.5 ± 4.3	yes
13	G-A	m	49	9	0/4 (0)	--	no
14	GBM	f	81	4	7/8 (88)	168.5 ± 14.1	yes
15	GBM	f	64	10	8/8 (100)	125.5 ± 14	yes
16	GBM	f	46	17	8/10 (80)	83 ± 2.4	no
17	F-A	f	38	56	0/6 (0)	--	no
18	GBM	m	64	26	6/7 (86)	134.5 ± 23.3	yes
19	G-A	m	34	54	5/6 (83)	326.5 ± 20	no
20	GBM	m	69	17	5/6 (83)	81.5 ± 3.4	yes
21	OA III	f	32	7	0/10 (0)	--	no
22	GBM	m	61	21	0/2 (0)	--	no
23	GBM	f	23	67	6/7 (86)	139 ± 8.6	no
24	GBM	m	60	28	6/8 (75)	126.5 ± 19	no
25	GBM	f	73	25	2/4 (50)	118.0 ± 40	no
26	GBM	f	66	8	5/8 (63)	111 ± 21	yes
27	GBM	m	56	35	2/4 (50)	68.5 ± 2.1	no
28	GBM	m	55	7	3/5 (60)	92 ± 12.9	no
29	GBM	f	74	8	3/4 (75)	102 ± 6	no

Abbreviations: GBM: Glioblastoma Multiforme, G-A: Gemistocytic Astrocytoma, F-A: Fibrillary Astrocytoma, OA III: Anaplastic Astrocytoma grade III, f: female, m: male.

**Figure 1:**
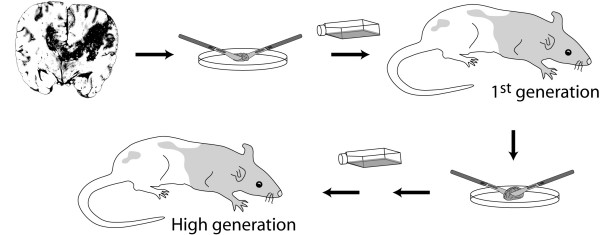
**Experimental design**. Biopsy tissues from human gliomas are minced into small fragments that are subsequently cultured in medium. The tumour fragments remodel and form small tumour spheroids, usually within 1-2 weeks. These spheroids are stereotactically implanted in the nude rat brain, and form xenograft tumours (1^st ^or low generation) that are harvested and processed similarly to the biopsy tissue. The resulting spheroids are passaged onto a new generation of rats and the previous steps can be repeated to passage the tumour for several generations.

**Figure 2:**
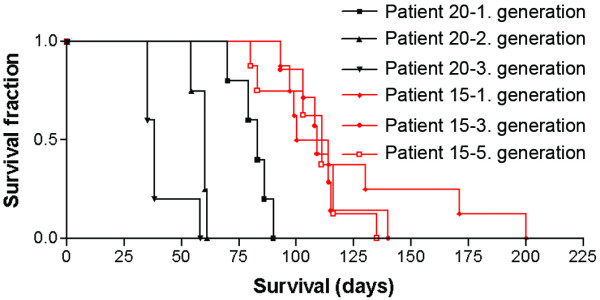
**Survival as a function of tumour passaging *in vivo***. Shown are survival data for nude rats xenografted with GBM-biopsy tissue that were passaged *in vivo *for several generations. The patient number refers to cases listed in Table 1. For rats xenografted with tumour tissue from patient 20, survival was significantly shorter with passaging for 3 generations (p = 0.0029). For rats xenografted with tissue from patient 15, survival also decreased with passaging, although this was not significant (p = 0.69).

For 11 GBM xenografts, tumour tissue was harvested from the rats and cultured *in vitro *similar to the human biopsies. The resulting tumour spheroids were re-implanted into a new generation of nude rats and serially passaged for up to 19 generations (Figure 1). Once biopsy spheroids had engrafted, the take rate remained close to 100% at all subsequent passages. The time from operation to onset of symptoms was recorded, and became gradually shorter with passaging (Figure 2). The difference in survival between first (low) and high generation was significant for three of the biopsies.

### Tumour histology

**Figure 3:**
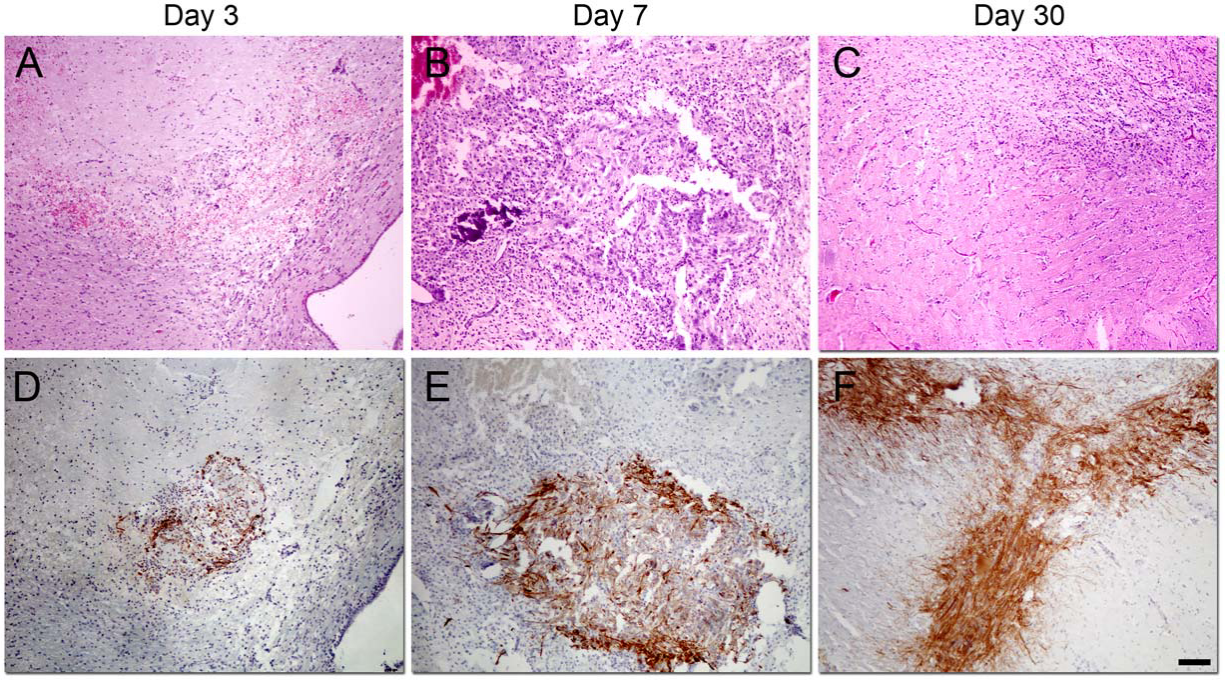
**Xenograft tumour histology in initial stages of tumour growth**. Growth of 1^st ^generation tumours 3 (A), 7 (B) and 10 days (C) following implantation of patient biopsy spheroids, H/E staining. Shown are the implantation site (A, B) and contralateral hemisphere (C). Corresponding sections were also stained with a human-specific antibody against nestin (D, E, F). Scale bars = 100 μm

In order to investigate the growth pattern in the initial phase after implantation we harvested 3 xenograft tumours at each time point, after 3, 7 and 30 days, (Figure 3). The tumours were H/E stained and neuropathologically examined. At 3 days, the implantation site appeared hypocellular in the center with cellular infiltration towards the periphery accompanied by microhemorrhages (Figure 3A). At the second time point, a small hypercellular tumour mass became visible at the implantation site. This lesion exhibited a gradual transition to the surrounding brain, and the haemorrhages at the periphery were smaller (Figure 3B). After 30 days, there was no longer a distinct lesion at the implantation site. Instead, there was a diffuse spread of tumour cells infiltrating cortical areas and white matter tracts, including the contralateral hemisphere (Figure 3C). Since our previous work has shown that the xenografted tumours express nestin we stained the corresponding sections with a human-specific antibody against nestin to distinguish tumour cells from the surrounding host cells (Figure 3D-F). This showed few immunopositive cells 3 days after implantation suggesting that the majority of cells are unable to adapt in the new environment (Figure 3D). After 7 days, more cells were visible, suggesting ongoing proliferation, and these cells were mostly localised to the implantation site (Figure 3E). Nestin staining of tumour sections at day 30 confirmed the H/E staining, demonstrating extensive tumour cell spread (Figure 3F).

**Figure 4:**
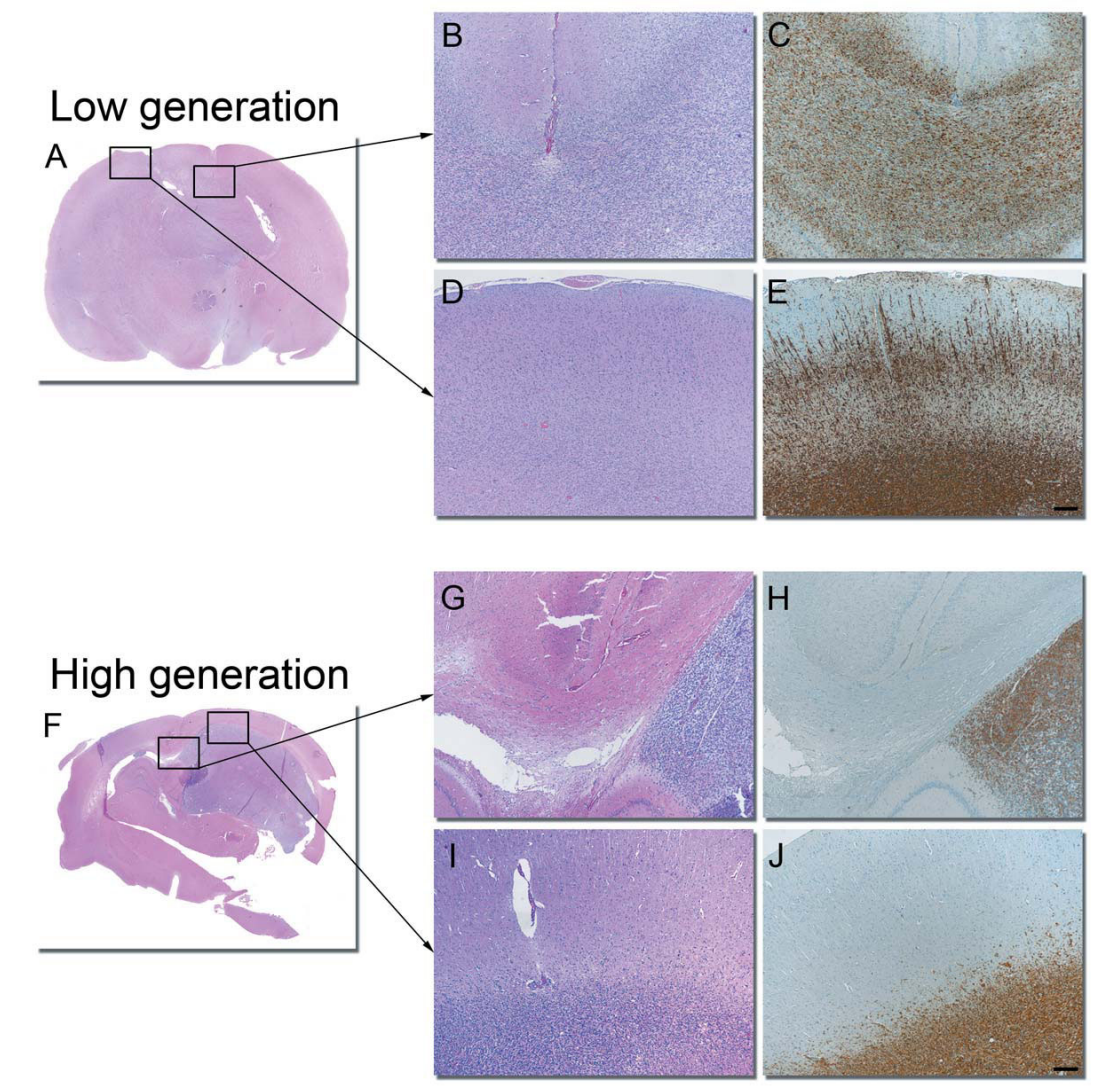
**Xenograft tumour histology in early and late generations**. Growth of 1^st ^(A, B, C, D, E) and 10^th ^generation (F, G, H, I, J) tumours in different areas of the nude rat brain. Corpus Callosum; H/E staining (B, G) and nestin staining (C, H), Cortex; H&E (D, I) and nestin (E, J). Scale bars = 250 μm.

The majority of the tumours was harvested at the onset of symptoms and was subsequently H/E stained (Figure 4). Thus, we also used a human-specific nestin antibody to get a precise view of tumour cell spread in the terminal stage (Figure 4). In the first generation, the tumours grew highly invasive with tumour cells migrating along white matter tracts and were also invading cortical structures. Glioma cells infiltrated the corpus callosum extensively towards the contralateral hemisphere (Figure 4A, B, C). Tumour cells were also observed infiltrating the cortex (Figure 4A, D, E), subventricular zone, and the basal ganglia. The tumour cells were found in both hemispheres of all the animals and in more than two cerebral lobes in most animals. Moreover, microvascular proliferations and necroses were absent in most of the first generation tumours. However, a subset of tumours showed these features already in the first or second generation (data not shown). Culture time of the biopsy spheroids did not impact on tumour histology in these experiments as spheroids cultured for both 3 days and 60 days grew highly invasive.

**Figure 5:**
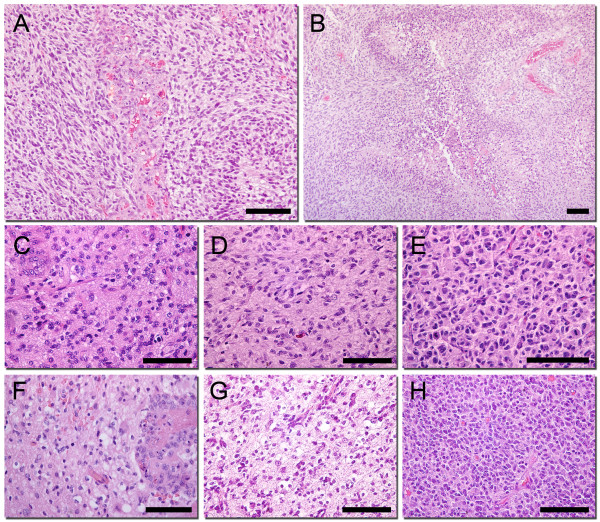
**Comparison of tumour cell morphology between patient and xenograft tumours**. (A) Glomeruloid microvascular proliferations in a high generation tumour, H&E staining. (B) Palisading necrosis in a high generation tumour, H&E staining. Patient biopsies (C, F) with corresponding low (D, G) and high generation xenografts (E, H), H&E staining. Scale bars = 100 μm

Successive passaging for 3-4 generations resulted in a gradual onset of angiogenesis and tumours that were both invasive and highly vascular. These tumours exhibited a typical GBM vasculature, with irregular dilated vessels, microvascular proliferations and glomeruloid tufts (Figure 5A). Furthermore, necrotic areas with typical pseudopalisading were present (Figure 5B). Altogether, the high generation tumours fulfilled all diagnostic criteria for GBMs. Although there was still a diffuse transition from the tumour to the surrounding host tissue, invasion into distant areas of the brain was less extensive compared to low passages. Extended passaging beyond 4 generations resulted in progressively less invasive tumours that displayed a more demarcated transition towards the surrounding host tissue (Figure 4F, G, F, H, I and 4J).

**Figure 6:**
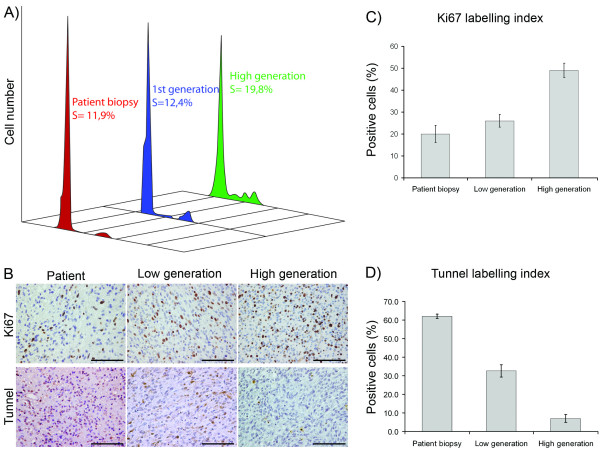
**Higher growth rates after passaging are mediated by increased tumour cell proliferation and reduced tumour cell death**. S-phase fraction in a patient biopsy and the resulting xenograft tumours at various stages of passaging (A). Ki67 and Tunnel staining of corresponding tumour biopsies (B), with quantification of their labelling indexes (C) and (D), respectively. Scale bars = 100 μm.

The morphology of the tumour cells from low and high generation GBM xenografts was compared to that of the cells in the patient biopsies at high magnification. In the first generation, the tumours were hypercellular, displaying moderate nuclear atypia as well as mitotic figures (Figure 5D, G). The nuclei of the tumour cells were mostly round to oval and similar to those observed in patient biopsies (Figure 5C, F). The cytoplasm of tumour cells was eosinophilic and showed fibrillary processes, a characteristic feature of gliomas in patients. The high generation tumours were highly cellular and the nuclei of tumour cells became more polymorphic and hyperchromatic compared to the first generation and patient-derived tumours. They often contained prominent nucleoli. The cytoplasm was less fibrillary and more circumscribed (Figure 5E, H). In addition, there were more mitotic figures, which lead us to specifically assess the growth kinetics of these tumours (Figure 6).

Thus, low generation tumours recapitulated the invasive growth of parental tumours and showed a similar cellular morphology and proliferation. In contrast, high generation tumours demonstrated all hallmarks of GBM growth, but the cells appeared more transformed and the mitotic index was higher compared to their parental tumours.

### Assessment of tumour growth by flow cytometry and immunohistochemistry

Since the overall tumour growth reflects the net balance between cell proliferation and death, we specifically assessed these parameters (Figure 6). Flow cytometry analysis showed that the S-phase fraction increased with passaging (Figure 6A). While the S-phase fraction in 1st generation tumours was 12.4% and close to the value estimated for the human biopsy of 11.9%, the S-phase fraction in the high generation tumours was 19.8%. Likewise, there was no significant differences in Ki67 labelling index (LI) between the low generation and the human biopsy derived tumours (26% and 20%, respectively, Figure 6B and 6C). However, LI was significantly higher in high generation tumours (49%) compared to the human biopsies and the low generation tumours (p = 0.0003 and p = 0.012, respectively). Thus, these findings corroborated the flow cytometry analysis to provide evident for greater cellular proliferation in the high generation tumours.

Moreover, we performed tunnel staining to compare apoptosis in the different tumour types (Figure 6B and 6D). Here, cell death levels were highest in the human tumour samples and showed a significant decline with passaging (p = 0.0008 and p = 0.037, respectively).

### MRI assessment of tumour growth

**Figure 7:**
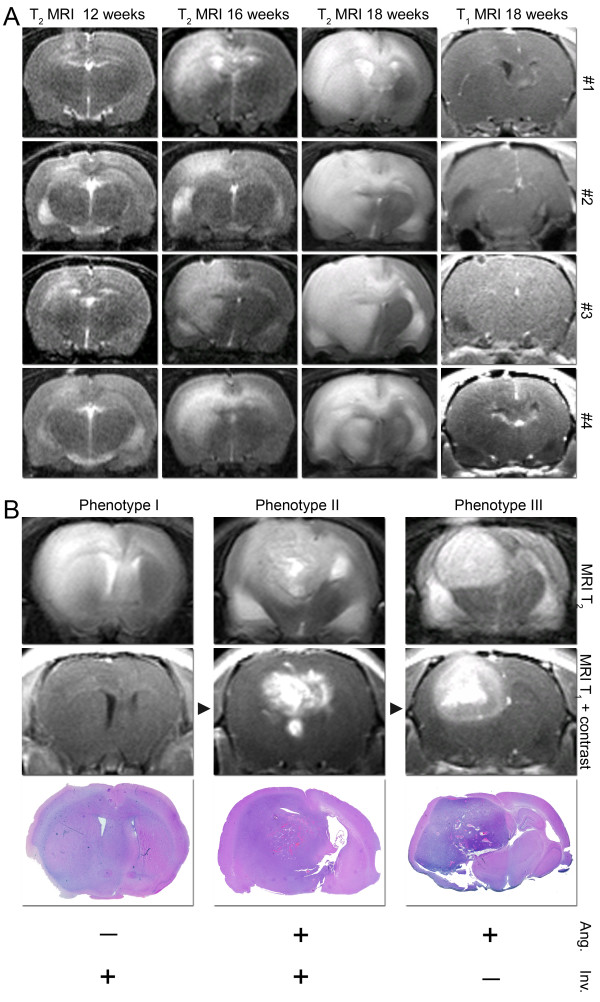
**MRI monitoring shows that brain tumour growth can be standardised and that the phenotypes are modulated with passaging *in vivo***. Coronary MRI scans showing tumour growth in four rats grafted with tissue from the same patient biopsy. Shown are T_2 _sequences at 3 different time points as indicated, while the right column present T_1 _images after administering contrast agent (A). MRI scans demonstrating different brain tumour phenotypes (B), displaying angiogenic and invasive growth as indicated (bottom). Corresponding H/E stained tumour sections are shown below the MRI panels. Extended passaging provide 3 distinct phenotypes that represent different combinations of invasive and angiogenic growth patterns.

The animals were monitored at various time points during the *in vivo *passaging by MRI using standard dynamic contrast enhanced clinical sequences. These included T1 with and without contrast agent injection for delineation of tumour volumes, as well as assessment of blood brain barrier function (Table 2 and Figure 7). T2 sequences were employed for visualising oedema and accumulation of cerebral spinal fluid (Figure 7). Longitudinal MRI revealed that the tumour growth characteristics were highly reproducible (Figure 7A). In the first generation tumours, the lesions appeared poorly delineated on T1 sequences, while secondary changes such as hydrocephalus and shift of midline structures (Table 2 and Figure 7B) indicated the presence of an expansive lesion. Contrast enhancement was usually not evident, while small focal regions of contrast leakage were detected in some tumours. A diffuse high signal intensity area was visualised with T2 sequences 8-10 weeks post implantation that increased gradually in size to occupy both hemispheres at the terminal stages. MRI scans of tumours from subsequent generations revealed a gradual onset of contrast enhancement up to 4th or 5th generation, after which the tumours remained strongly contrast enhancing. However, with further passaging the tumours gradually displayed less infiltrative growth and became more circumscribed. Thus, passaging *in vivo *provided different combinations of invasive and vascular tumour phenotypes; 1) non-angiogenic and invasive tumours, 2) angiogenic and invasive tumours and 3) angiogenic and poorly invasive tumours (Figure 7B).

**Table 2:** MRI characteristics of xenograft tumours in low and high generation.

Radiological Feature	Low Generation	High Generation
Edema in tumour mass	15/15	8/8
Edema in contralateral hemisphere	15/15	3/8
Hydrocephalus	14/15	8/8
Contrast enhancement (T_1_)	4/15	8/8

## Discussion

Outcome and treatment has improved only marginally over the last decades for GBMs [22], and reflect a limited understanding of the mechanisms that regulate their biological behaviour. This again underscores the need for models that reliably mimic their invasive and vascular growth. Transgenic brain tumour models, involve germline or somatic genetic modifications that predispose animals to developing CNS tumours [23-25]. However, whereas these models have a limited number of defined genetic changes, human brain tumours are characterised by multiple genetic aberrations. Alternatively, tumour development can be induced by carcinogen exposure or established by implantation of [26,27] or xenogeneic [26] cancer cells or tumour biopsies [19,20]. While cell line based models often fail to mimic essential characteristics of brain tumours [4], some studies have reported growth of highly infiltrative tumours, following the implantation of human glioma biopsies in the nude rat brain [4,19,20]. Sakaria et al reported that the parent tumour's EGFR amplification status was preserved through subcutaneous passaging of the glioblastoma xenografts in nude mice [28]. Moreover, cell suspensions established from short-term *in vitro *cultures of flank xenograft tumours could subsequently be xenografted orthotopically. They successfully applied this approach to investigate how EGFR amplification status impacted on response to radiation therapy. Here, we report another approach passaging GMB xenografts intracerebrally, using nude rats as the host. Since tumour-host interactions are organ-specific, we believe that orthotopic passaging throughout the experiment avoids adaption towards a foreign niche, such as subcutaneous tissue. Furthermore, the somewhat larger size of the rat brain makes it possible to conduct certain experiments, such as convection-enhanced delivery from subcutaneously implanted pumps, which are not easily performed in mice. Moreover, we implant spheroids initiated from the tumours which also contain ECM components and stromal cells within the tumour environment, whereas this stromal compartment is disrupted when cell suspensions are used. Previously, we have also shown that first generation tumours derived from GBM biopsies often exhibit angiogenesis-independent growth while passaging is accompanied by the onset of angiogenesis, producing highly vascular tumours [20,29,30]. Moreover, gene expression profiling and protein arrays demonstrated that this phenotypic shift coincided with alterations in signalling pathways [20]. Whereas components of the Wnt, PI3K, and NF-kβ signalling pathways were overexpressed in the invasive first-generation tumours compared with the high-generation tumours, the Ras signalling pathway was up-regulated in the high generation tumours.

However, although this model mimics essential brain tumour phenotypes, its implementation require that it provides sufficiently reproducible data as well. Whereas many aspects related to the use of cancer cell lines can be standardised, outcome parameters such as tumour growth and survival data from cell line experiments may still vary. In our series, average survival was 118 days, whereas the average standard error of the mean (SEM) was 9.3 days, which compares well to other cell line based studies [31-33]. Moreover, 24 of 25 (96%) GBM biopsies produced tumours in 137 of 171 (80%) nude rats. These findings are similar to a previous smaller study in which 5 of 6 (83%) of the GBM biopsies produced tumours in 38 of 47 (81%) nude rats [19]. This is also in line with numerous studies using primary biopsies from other subtypes that provide high tumour take rates [3,15-18,34-37]. The low take rates we observed with biopsies from grade II and III tumours may be due to a lack of adaptability to the rat brain environment. However, while grade II gliomas may appear radiologically unchanged for several years in the patient, the life span of nude rats is in the range of 1,5 -2 years under specific pathogen free conditions [38]. Therefore, the low take rates may simply reflect that the time required to develop clinically manifest tumours from low grade glioma biopsies exceeded the life span of the hosts. Although the number of lower grade tumours was small in this study, these findings still question the use of this model system to study such tumours. In summary, these findings demonstrate that the large majority of GBMs engraft in nude rats with a high take rate, and mediate a reproducible disease course if experimental parameters are standardised.

Furthermore, MRI provided information about essential aspects of tumour growth such as tumour volume, contrast enhancement, invasiveness, shift of midline structures, surrounding oedema as well as hydrocephalus. Based on these features, MRI reliably distinguished the different tumour phenotypes identified by histological examination. Thus, MRI with clinical sequences can be used in used in conjunction with this model to distinguish the different tumour phenotypes by and monitor growth by imaging *in vivo*.

Histological analysis at day 3 after implantation revealed that the implantation site contained few cells in the centre, but with cellular infiltration and microhemorrhages at the periphery. This picture may reflect initial reactive changes following implantation, where most tumour cells fail to adapt to the new environment and die. Accordingly, the small tumour visible in the implantation site after 7 days reflects a regrowth of the tumour cells that survive the initial phase. The extensive spread of tumour cells after 30 days suggest that activation of pro-invasive programs is an integral part of the adaptive behaviour needed to sustain growth during the early phases.

In the late stages, at the onset of symptoms, the first generation tumours recapitulated the invasive features of the parent tumours like previously reported, but were largely devoid of angiogenesis and necrotic areas. Moreover, tumour cells were found in more than two cerebral lobes and in the contralateral hemisphere. The morphology of tumour cells at high magnification was similar to that observed in patient biopsies.

In contrast, high generation tumours fulfilled all diagnostic criteria of human GBMs, but grew less invasive. The tumours had a higher cell density and the tumour cells displayed a more malignant phenotype compared to low generation tumours and patient biopsies with large, polymorphic nuclei and a less fibrillary, but more circumscribed cytoplasm. Immunohistochemistry also showed that the Ki67 labelling was higher in the tumour xenografts than in the patient biopsies, and increased with passaging. Moreover, tunnel analyses shows that cell death rates declined with propagation *in vivo*, pointing to an additional mechanism for the increased overall growth rates that we observed in higher generation tumours. Thus, the faster growing high generation tumours result both from increased gain due to higher cell proliferation, as well as reduced cell loss due to less apoptosis.

It is an important question whether this progression seen in the rat is also the course of primary GBMs in the patients. It is conceivable that the initial GBM in the patient is growing as an invasive lesion without angiogenesis. GBMs are mostly diagnosed at late stages, when the tumours have reached a certain size causing symptoms and changes in MRI due to mass effects, angiogenesis with breakdown of the blood-brain-barrier and necrosis. Thus, early stages are not easily detectable on contrast enhanced sequences, due to lack of these features. While we previously demonstrated that passaging the tumours for 4-5 generations was accompanied by an onset of angiogenesis [20], this study includes biopsies that have been passaged for up to 19 generations. MRI imaging of the animals and histological examination of the tumours revealed that extended passaging was accompanied by a gradual loss of their invasive phenotype. Thus, *in vivo *passaging provides a model for modulating essential brain tumour phenotypes. Since these different phenotypes can be derived from the same patient, variation in genetic background due to inter-individual differences is eliminated. Further, systematic comparisons of angiogenic and non-angiogenic tumours that are both invasive and otherwise similar may help identify genes and proteins critical to the onset and maintenance of brain tumour angiogenesis. Using the same strategy on invasive and non-invasive tumours can help to identify invasion-related genes and proteins [29]. We therefore believe this model provides a powerful tool do dissect mechanisms that mediate angiogenesis and invasive growth.

## Conclusions

In conclusion, our data demonstrate that xenograft tumours representative of the parent tumour can be established in nude rats with a high take rate and a reproducible disease course. Furthermore, MRI using standard clinical sequences provides useful information regarding critical GBM phenotypes involved in tumour progression. Studies at various time points as well as at different stages of passaging suggest that the adaptive behaviour of these tumours display to main phases; one initial phase relying on pro-invasive programmes, and one later stage characterised by angiogenesis, increased cell proliferation and reduced apoptosis, leading to increased overall growth rates. In humans, this model may have a special relevance to early stages of tumour growth, prior to onset of symptoms and diagnosis.

## Competing interests

The authors declare that they have no competing interests.

## Authors' contributions

JW carried out tissue culture, animal experiments and MRI scanning, participated in study design and manuscript preparation. HM, PCH, NB, HG and SW carried out histological examination and immunohistochemistry staining. PS helped manuscript editing. XL and PZ helped animal experiments. MC performed tunnel staining. RB helped in flow cytometry, participated in study design. PE helped statistical analysis, participated in study design and manuscript preparation. All authors read and approved the final manuscript.

## Pre-publication history

The pre-publication history for this paper can be accessed here:

http://www.biomedcentral.com/1471-2407/9/465/prepub
